# Radiation response assessment of organoids derived from patients with pancreatic cancer

**DOI:** 10.1016/j.ctro.2024.100829

**Published:** 2024-07-27

**Authors:** Iris W.J.M. van Goor, Leon Raymakers, Daan S.H. Andel, Lodewijk A.A. Brosens, Onno Kranenburg, Jeanette H.W. Leusen, Gert J. Meijer, I. Quintus Molenaar, Hjalmar C. van Santvoort, J.H. Wilfred de Vries, Andre J.M. Wopereis, Martijn P.W. Intven, Lois A. Daamen

**Affiliations:** a Department of Surgery, Regional Academic Cancer Center Utrecht, Utrecht University, University Medical Center Utrecht Cancer Center & St. Antonius Hospital Nieuwegein, Utrecht, the Netherlands; b Department of Radiation Oncology, Regional Academic Cancer Center Utrecht, Utrecht University, University Medical Center Utrecht Cancer Center & St. Antonius Hospital Nieuwegein, Utrecht, the Netherlands; c Center for Translational Immunology, UMC Utrecht, Utrecht University, Utrecht, the Netherlands; d Department of Surgical Oncology, Lab of Translational Oncology, UMC Utrecht Cancer Center, University Medical Center Utrecht, Utrecht University, Utrecht, the Netherlands; e Department of Pathology, Regional Academic Cancer Center Utrecht, Utrecht University, University Medical Center Utrecht Cancer Center & St. Antonius Hospital Nieuwegein, Utrecht, the Netherlands; f Imaging Division, University Medical Center Utrecht Cancer Center, Utrecht University, Utrecht, the Netherlands

**Keywords:** Pancreatic ductal adenocarcinoma, Radiation, Radiotherapy, Patient derived organoids, Sensitivity, Response, Dose–response correlation

## Abstract

**Background:**

The effectiveness of radiotherapy for pancreatic cancer is debated. Patient-derived organoids (PDOs) already mimicked clinical radiation response in other cancer types, which could be valuable in pancreatic cancer as well. This study aimed to investigate whether PDOs can be used to model RT response in pancreatic cancer and to explore the presence of a dose–response correlation.

**Methods:**

PDOs derived from two pancreatic cancer patients (HUB-08-B2-022A and HUB-08-B2-026B) were irradiated with doses ranging from 0 to 40 Gray. Viability assessments were conducted after seven and 10 days by measuring ATP-levels. Results were normalized, defining the viability at 0 Gray as 100 % and an absolute viability of 0 as 0 %. The relative area under the curve (rAUC) was calculated (0 = total sensitivity, 1 = total resistance).

**Results:**

With a readout time of seven days, both HUB-08-B2-022A and HUB-08-B2-026B exhibited viability above 50 % at the highest dose of 12 Gy (rAUC of 0.79 and 0.69, respectively). With a readout time of 10 days, both PDOs showed a dose–response relation although HUB-08-B2-022A was more sensitive than HUB-08-B2-026B (rAUC of 0.37 and 0.51, respectively). Increasing the radiation dose to 40 Gy did not further affect viability, but the dose–response relation remained present (rAUC of 0.13 and 0.26, respectively). In the final experiment with a readout time of 10 days and a maximum dose of 14 Gy, the dose–response correlation was paramount in both PDOs (rAUC 0.28 and 0.45, respectively), with HUB-08-B2-022A being most sensitive.

**Conclusions:**

In this setup, both pancreatic cancer PDOs showed an irradiation dose–response correlation. These preliminary findings suggest that pancreatic cancer PDOs are suitable for assessing radiation response *in vitro*. Further experiments are needed to eventually simulate treatment responses to personalized treatment strategies.

## Highlights

• Pancreatic cancer patient derived organoids (PDOs) can model radiation response.
• Both PDOs were sensitive to irradiation, although heterogeneity in response was found.
• Sufficient time until readout (i.e., 10 days) is crucial for proper evaluation of results.

## 1 Introduction

Pancreatic cancer is one of the most lethal cancers worldwide.[1] Until recently, radiotherapy (RT) did not play a major role in pancreatic cancer treatment. Ablative radiation doses with conventional computer tomography (CT) guided systems were impeded due to poor visibility of pancreatic tumors and surrounding radiosensitive organs at risk (OAR). Furthermore, movement of the pancreas caused by peristalsis and breathing complicated precise dose delivery.[2], [3] The landscape has changed with the emergence of magnetic resonance guided RT (MRgRT). Enhanced soft tissue contrast with MR improves tumor and OAR visibility. In addition, daily online adaptive treatment planning and tumor gating through continuous cine MR-images increases precise dose delivery to the target area, while sparing the OAR.[4], [5], [6], [7], [8], [9], [10] These advantages enable irradiation of pancreatic lesions with higher biologically equivalent doses, potentially increasing treatment efficacy.[11].

However, awaiting results from ongoing randomized trials, evidence about the effectiveness of RT for pancreatic cancer is currently lacking. Consequently, discussion remains whether pancreatic cancer is sensitive to RT, and whether an increased radiation dose also has an increased effect on the viability of the tumor. Yard et al. have irradiated 2-dimensional (2D) cell lines of various types of cancers with doses ranging from 0 to 10 Gray (Gy).[12] We compared the discovered treatment effects, measured by the relative Area Under the Curve (rAUC), between pancreatic cancer and other commonly with RT treated cancers such as esophageal, lung, and breast cancer by a student's *t*-test. No statistically significant differences between pancreatic cancer and other cancers were found. This suggests that pancreatic cancer cells have similar sensitivity to radiation as cells of other cancer types. Nonetheless, the use of 2D cell lines has shortcomings, as it does not support growth of untransformed, neoplastic pancreatic cells.[13].

Patient-derived organoids (PDOs), on the other hand, can mimic the histological and phenotypical heterogeneity of the original tumor while genetic characteristics stay preserved.[13] Yao et al. and Ganesh et al. demonstrated that in rectal cancer patients, PDOs were able to predict clinical (chemo)radiation response.[14], [15] Theoretically, PDOs could be useful to study radiation response in pancreatic cancer as well. Research on RT sensitivity of pancreatic cancer using PDOs is, however, scarce. The two studies investigating this topic concluded differently on pancreatic cancer’s radiation sensitivity: one study reported limited sensitivity of pancreatic cancer PDOs at clinically relevant radiation doses, while the other study concluded that all PDOs exhibited relevant responses to these doses.[16], [17] An important difference between the studies was the variation between their experiments’ readout times, which could explain the different conclusions about sensitivity.

The aim of this study was to investigate whether PDOs can be used to model RT response in pancreatic cancer and to explore the presence of a dose–response correlation.

## 2 Materials and methods

Two patient-derived pancreatic cancer organoid lines from treatment naïve pancreatic cancer tissue obtained from the pancreatic resection specimen were generated by the Hubrecht Organoid Foundation (HUB, https://www.hub4organoids.eu). Derivation of the pancreatic cancer PDOs from the surgical specimen, as well as the organoid passaging and their histological and genetic characteristics (Supplementary Table 1) were previously described by Driehuis et al.[13], [18] Informed consent for organoid derivation and analysis was obtained according to the biobanking protocol HUB-Cancer TcBio#12-09.

### 2.1 Patient characteristics

The nationwide Pancreatic Cancer Recurrence Database (NCT04605237) was used to obtain the patients’ surgical, histological, treatment, follow-up, and survival data. Tumor (T) stage, nodal (N) status, and TNM-status were defined according to the eighth edition of the American Joint Committee on Cancer TNM guidelines.[19] Resection margins were declared microscopically positive (R1) when tumor cells were present within 1 mm of the closest resection margin, apart from the anterior surface.[20] Overall survival (OS) was defined as the time between the date of surgery and the date of death from any cause. Disease-free survival (DFS) was defined as the time between the date of surgery and the date of recurrence diagnosis.

### 2.2 Irradiation assays

**Fig. 1:**
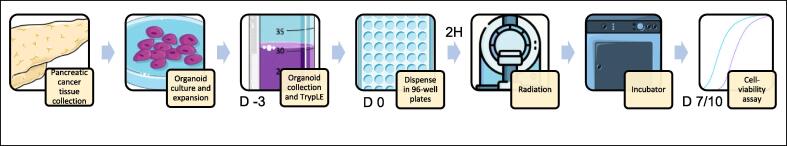
Experimental set-up of *in vitro* drug screens on Patient Derived Organoids (PDOs) derived from pancreatic cancer. PDOs were derived from pancreatic cancer tissue derived from patients during their resection. They were cultured in Matrigel, immersed in organoid culture medium. Three days prior to the drug screens, the PDOs were enzymatically made single cell. On the day of the experiment, they were plated on 96-well plates after which they were irradiated with doses ranging from 0–40 Gray, depending on the set-up. Seven to 10 days later, viability was assessed. D day(s); H hours.

Three days prior to the irradiation assays, the PDOs were enzymatically made single cell using TrypLE™ (Gibco™, ThermoFisher Scientific, Paisley, United Kingdom) (Fig. 1). A 40 µm filter was used to dispense the cells in 6-well plates (Costar® 3764; Corning, New York, USA).

At the day of the irradiation, the PDOs were dissolved in organoid culture medium (either TM1 or TM2, Supplementary Table 2a-c).[13], [14], [15], [16], [17], [18] TM1 lacks EGF, whereas TM2 lacks Wnt and A83-01 which can influence the suitability of a medium for growth of a specific PDO. However, both PDOs in this study were able to grow in TM1 as well as TM2 medium.[18] According to their respective stock mediums, HUB-08-B2-022A was dissolved in TM2, while HUB-08-B2-026B was dissolved in TM1. At a concentration of approximately 1000 PDOs/µL, the suspension was dissolved in Matrigel® (356231; Corning, New York, USA) in a 1:3 ratio, leading to a final concentration of approximately 250 PDOs/µL. Next, 4 µL of this solution (containing 1000 organoids) was dispensed as droplets in each well of a 96-well plate. These plates were placed upside down in an incubator set at 37 degrees (Sanyo MCO-19AIC (UV) CO2 incubator, Sanyo Electric Corporation, Moriguchi, Japan) for 20 min. Organoid culture medium was added using a multichannel pipette and the plates were put back in the incubator for another 60 to 120 min. Subsequently, the PDOs were irradiated with a linear accelerator (linac, Synergy Versa HD, Elekta, Stockholm, Sweden) with a 6MV photon beam and a 20x20cm^2^ field size. The 96-well plate was irradiated from gantry angle 180 degrees with 190 Monitor Units (MU) per 2 Gy of absorbed dose. Twenty mm of RW3 solid water slab (PTW Freiburg) was used as build-up material. The isocenter was placed on top of the RW3 and below the 96-well plate. Polystyrene plates were placed between the radiation field and plate to establish photon scattering. Doses between 0–40 Gy were given, and each dose level was delivered on a separate 96-well plate. The plates then remained in the incubator for a set number of days (i.e. seven or 10 days), depending on the experiment.

After the incubation period, EVOS cell imaging System (Thermo Fisher Scientific) was used to assess the condition of the PDOs by visualizing their number, size, and overall morphology. Subsequently, adenosine triphosphate (ATP)-levels were assessed using a CellTiterGlo® 3D kit (Promega, Madison, Wisconsin, USA). In each well, 100 µL of CellTiterGlo® was added, after which the plate was shaken for five minutes using a Multidrop (ThermoFisher™ Scientific, Waltham, Massachusetts, USA) to ensure proper distribution of CellTiterGlo® in the well. Until readout, the plate was kept completely in the dark. Exactly 30 min after dispensing CellTiterGlo®, cell viability was measured on a Spectramax M5e reader (Molecular Devices, San Jose, California, USA). Viability assays were performed in sextuple.

The first experiment comprised of irradiation dose levels of 0–12 Gy, with a time until readout of seven days based on experience of our lab with measuring dose–response correlations in colorectal cancer organoids. A second experiment, using equal irradiation doses but with a prolonged time until readout of 10 days was conducted. Third, an experiment with 10 days until readout, but irradiation doses of 0–40 Gy was performed. Finally, an experiment with an irradiation dose of 0–14 Gy and a time until readout of 10 days was done. To confirm the results, the final experiment was repeated three times for reproducibility (See Table 1).

**Table 1:** Set-up of all experiments.

**Experiment**	**Dose level**	**Time until readout**
1	0–12 Gy	7 days
2	0–12 Gy	10 days
3	0–40 Gy	10 days
Final	0–14 Gy	10 days

### 2.3 Data analysis

Data retrieved from viability assays with the Spectramax reader was analyzed using Graphpad Prism. Viability was normalized, whereby the mean of six control wells (containing organoid solution which was not irradiated) per PDO was defined as 100 % viability, and an absolute value of 0 was defined as 0 % viability. It was reported with 95 % confidence intervals (95 % CI). A linear-quadratic dose–response model was fitted.[21] Outliers were detected and eliminated with a ROUT coefficient Q of 1 %. The hill slope was constrained to −1, and the S was set at being greater than 0.5.[22] Finally, the rAUC was calculated by dividing the observed AUC by the theoretical maximum AUC; yielding a number between 1 (very resistant) and 0 (very sensitive).

## 3 Results

### 3.1 Patient characteristics

HUB-08-B2-022A was derived from a 75-year-old female patient. She underwent a pancreaticoduodenectomy for a pancreatic head tumor of 30 mm. Lymphovascular and perineural invasion were present and positive microscopic resection margins were found (R1 direct). Five out of 13 obtained lymph nodes were tumor positive (N2). The patient did not receive adjuvant chemotherapy because of poor clinical condition. Sixteen months after surgery, the patient was diagnosed with local disease recurrence combined with lung and peritoneal metastases, for which the patient received best supportive care. Overall survival was 19 months. The second PDO, HUB-08-B2-026B, was derived from a 63-year-old male patient. He underwent a pancreaticoduodenectomy for a moderately/poorly differentiated pancreatic head tumor of 43 mm (T3). Lymphovascular and perineural invasion were present. Three out of 29 lymph nodes were tumor positive (N1), and tumor infiltration was found in the resection margin of the superior mesenteric vein (R1 direct). Nine weeks postoperatively, six cycles of adjuvant gemcitabine were started and completed as planned. Fifteen months after surgery, the patient presented with abdominal pain. A CT-scan revealed local disease recurrence and liver metastases. Because of poor clinical condition, best supportive care was given. Overall survival was 19 months.

Both PDOs had a cystic appearance when in culture (Supplementary Fig. 1).

### 3.2 Irradiation viability assays

**Fig. 2:**
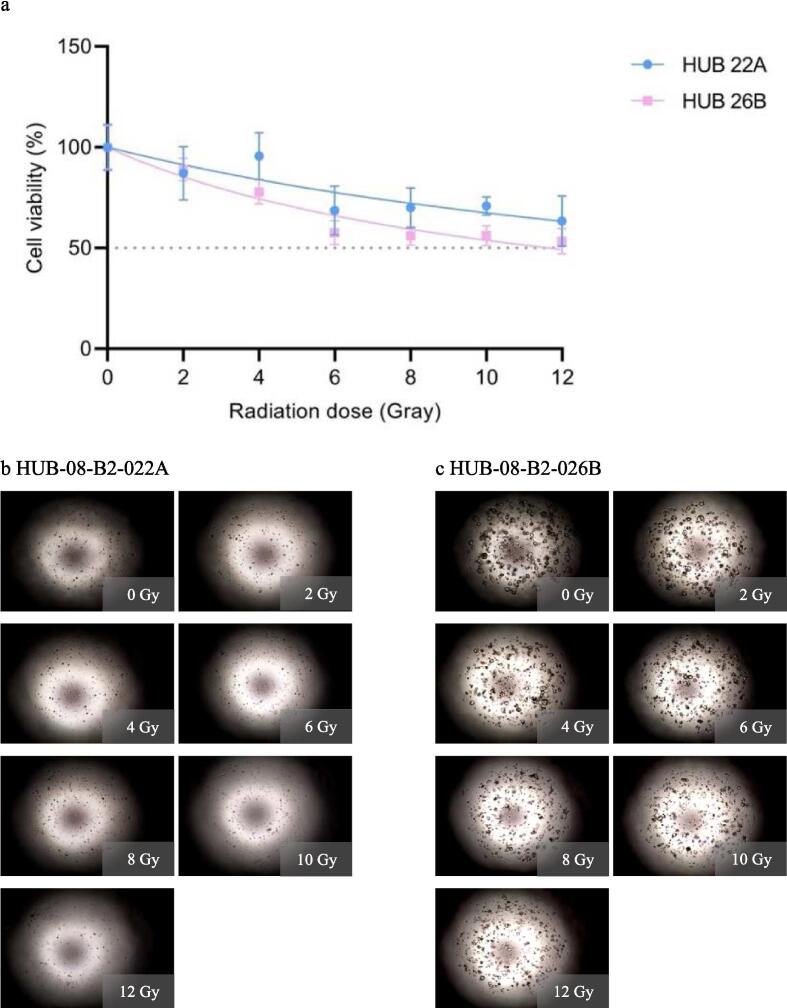
First radiation response assessment (0–12 Gray with seven days incubation time). a: Graph shows ATP viability assays (%) at seven days after receiving irradiation doses ranging from 0–12 Gray b: Cell imaging of HUB-08-B2-022A at seven days after receiving doses ranging from 0–12 Gray, c: Cell imaging of HUB-08-B2-026B at seven days after receiving doses ranging from 0–12 Gray.

Seven days after the first experiment with doses ranging from 0-12 Gy, both HUB-08-B2-022A and HUB-08-B2-026B maintained a viability of at least 50 %, even at the highest irradiation dose of 12 Gy. The rAUCs were 0.79 (95 % CI 0.73–0.85) and 0.69 (95 % CI 0.65–0.72) respectively. Corresponding images also showed a lack of response in both PDOs (Fig. 2).

**Fig. 3:**
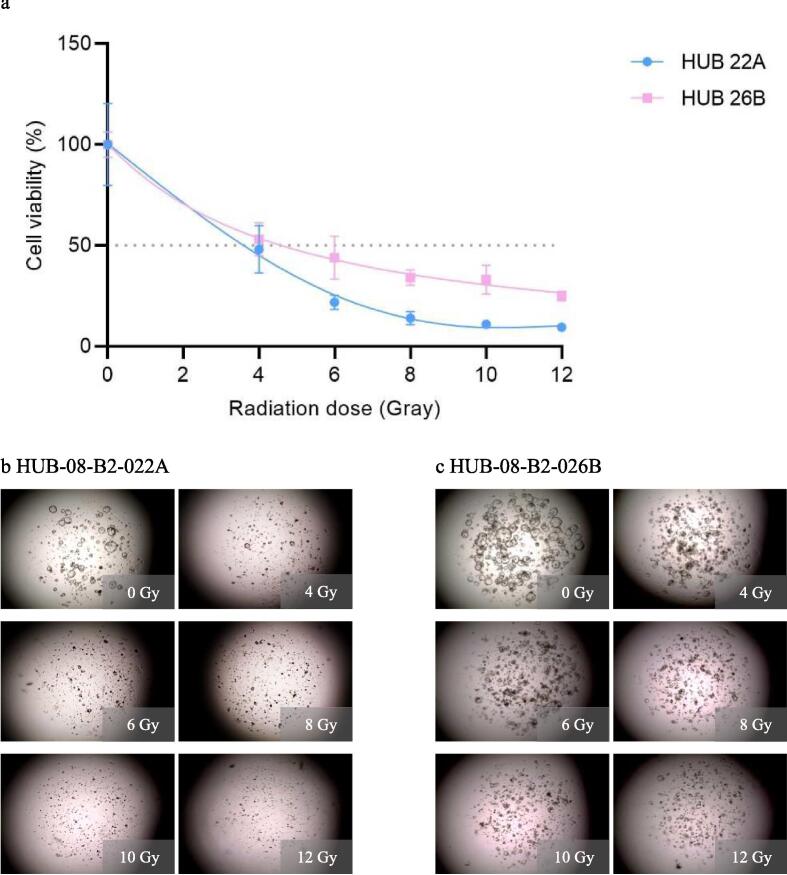
Second radiation response assessment (0–12 Gray with 10 days incubation time). a: Graph shows ATP viability assays (%) at 10 days after receiving irradiation doses ranging from 0–12 Gray b: Cell imaging of HUB-08-B2-022A at 10 days after receiving doses ranging from 0–12 Gray, c: Cell imaging of HUB-08-B2-026B at 10 days after receiving doses ranging from 0–12 Gray.

In the second experiment, viability was measured after 10 days while the irradiation dose range from the first experiment was maintained. HUB-08-B2-022A was more sensitive to radiation than HUB-08-B2-026B, with an rAUC of 0.37 (95 % CI 0.29–0.45) and 0.51 (95 % CI 0.46–0.55) respectively. With the increased time until readout of 10 days both PDOs now showed a clear dose–response correlation which was absent after seven days. On the corresponding images, it is also visible that HUB-08-B2-026B is less sensitive to RT as compared to HUB-08-B2-022A (Fig. 3).

**Fig. 4:**
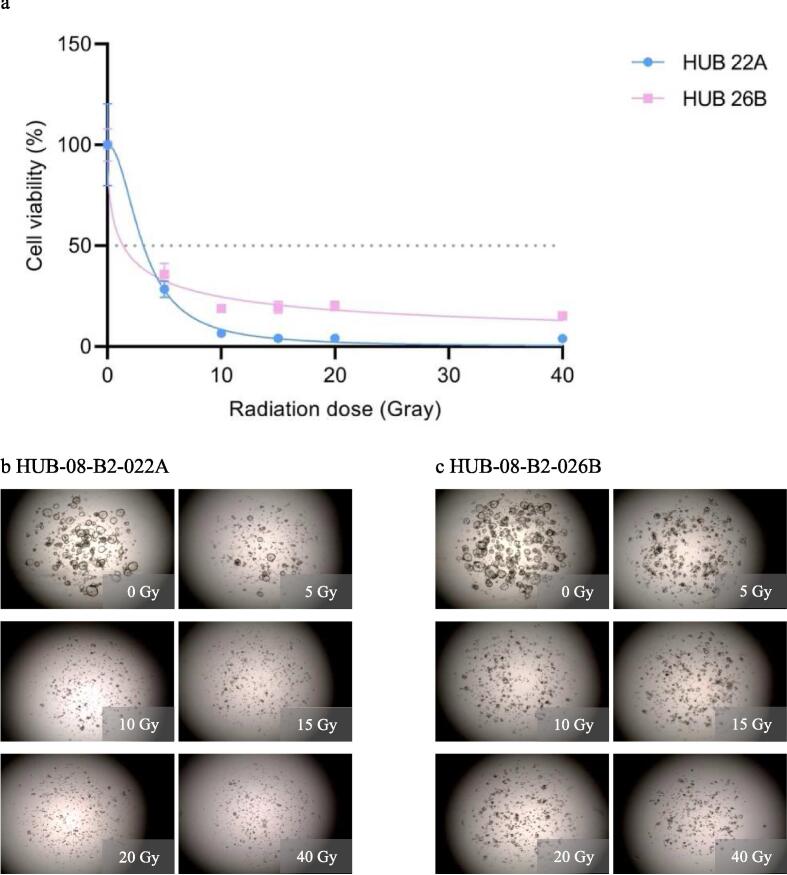
Third radiation response assessment (0–40 Gray with 10 days incubation time). a: Graph shows ATP viability assays (%) at 10 days after receiving irradiation doses ranging from 0–40 Gray b: Cell imaging of HUB-08-B2-022A at 10 days after receiving doses ranging from 0–40 Gray, c: Cell imaging of HUB-08-B2-026B at 10 days after receiving doses ranging from 0–40 Gray.

In the third experiment, the maximum irradiation dose was increased from 12 to 40 Gy while the time until readout was kept at 10 days. Results were comparable to those of the second experiment: the dose–response correlation is present with HUB-08-B2-022A being more sensitive (rAUC of 0.13 (95 % CI 0.11–0.16)) than HUB-08-B2-026B (rAUC of 0.26 (95 % CI 0.24–0.28)). Viability in both PDOs did not further decrease when irradiated with doses ranging from 10–40 Gy (Fig. 4).

**Fig. 5:**
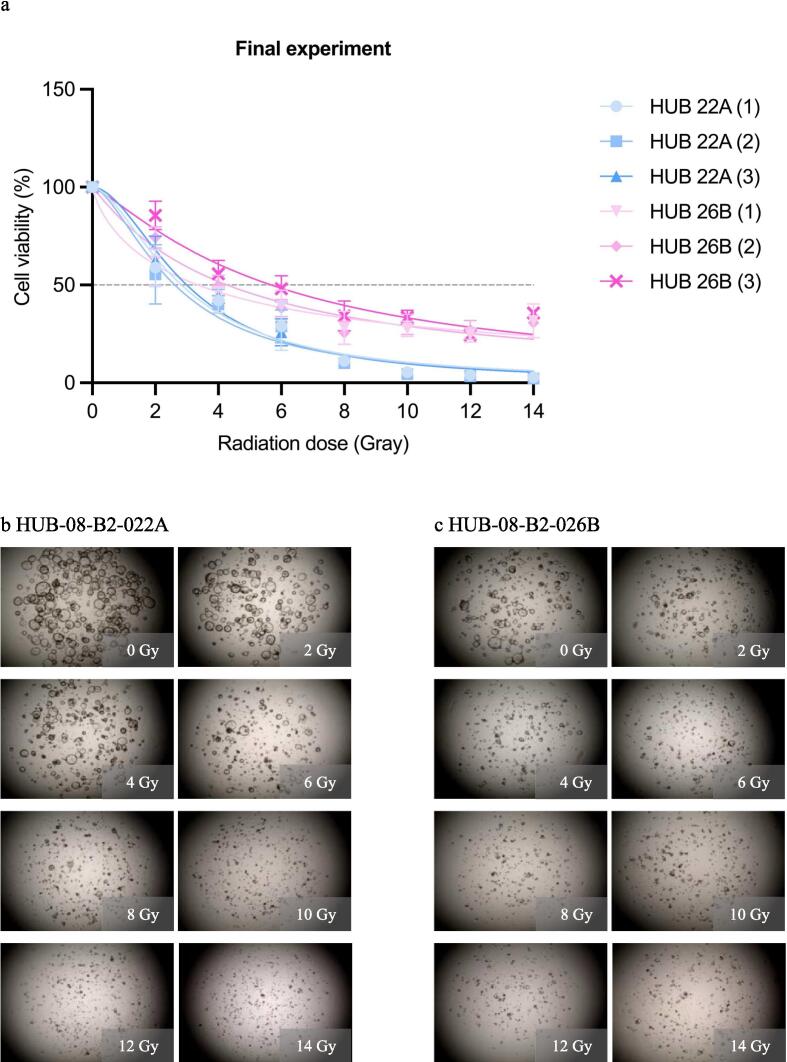
Final radiation response assessment (0–14 Gray with 10 days incubation time). a: Graph shows ATP viability assays (%) at 10 days after receiving irradiation doses ranging from 0–14 Gray b: Cell imaging of HUB-08-B2-022A at 10 days after receiving doses ranging from 0–14 Gray, c: Cell imaging of HUB-08-B2-026B at 10 days after receiving doses ranging from 0–14 Gray.

In the final experiment, a dose range of 0–14 Gy was given while results were still assessed after 10 days. HUB-08-B2-022A was more sensitive than HUB-08-B2-026B, with an rAUC of 0.28 (95 % CI 0.25–0.31) versus 0.45 (95 % CI 0.42–0.48), respectively. Gradual PDO response was also seen from the lowest to the highest RT dose, with consequent viability in the highest three doses (Fig. 5).

## 4 Discussion

This study confirms that PDOs can be used to model radiation response in pancreatic cancer, which has been questioned by contradictory findings of two previous studies. While absent with a time until readout of seven days, both PDOs showed an irradiation dose–response correlation between doses of 0 to 14 Gy at 10 days after irradiation. This suggests that pancreatic cancer is sensitive to radiation and emphasizes that sufficient time to readout is crucial for proper evaluation of results.

Two studies previously investigated irradiation sensitivity of pancreatic cancer PDOs. Nicosia et al. studied the effect of RT in six PDOs derived from treatment naïve pancreatic cancer patients. A maximum dose of 12 Gy was given, followed by short-term viability assays after 72 h.[16] Irradiation response varied between the PDOs. Two PDOs showed close to no response, even at the highest dose level of 12 Gy, and only one PDO showed a viability below 40 % after irradiation with doses of 10 and 12 Gy. The authors concluded that the PDOs showed limited sensitivity to RT. However, the time until readout was only 3 days. In our study, both PDOs still had more than 50 % viability after seven days, while they did show a clear dose–response correlation after 10 days. Naumann et al. assessed the effect of RT on four pancreatic cancer PDOs. Viability of the PDOs was assessed at day six, nine, and 13 after receiving a maximum dose of 12 Gy.[17] In these experiments, further decrease in viability was seen in case of a prolonged readout time. When assessed at day 13, all PDOs showed a dose–response correlation with a viability of less than 25 % at 12 Gy. This might be explained by the fact that pancreatic cancer cells are slowly proliferating, which also might cause a delayed treatment response. In addition, the main therapeutic mechanism of RT involves inducing mitotic catastrophe, which enhances late cell-death.[23] Therefore, to visualize the presence of the dose–response correlation, it seems paramount to incorporate sufficient time until readout to obtain valid results. However, the most appropriate timing is yet to be determined. Furthermore, Nicosia et al. and Naumann et al. irradiated with a maximum of 12 Gy in their studies. Although five out of six PDOs exhibited some dose–response correlation in the study performed by Nicosia et al, only two PDOs reached a viability of less than 50 % at the maximum dose of 12 Gy.[15] However, drawing conclusions about whether a maximum dose of 12 Gy is sufficient to expose the hypothetical dose–response correlation based on this study is challenging because of the short readout time. In contrast, Naumann et al. assessed the impact after a maximum time of 13 days after RT, which we presume to be sufficient time until readout. With a maximum dose of 12 Gy, all four organoids demonstrated a clear dose–response correlation.[17] These results correspond to our findings and indicate that an irradiation dose of 12 Gy is adequate for detecting the dose–response effect in PDOs of pancreatic cancer. Additionally, we investigated the impact of radiation doses up to 40 Gy and observed that there was no additional effect on viability beyond a dose of approximately 12 Gy, suggesting a plateau phase was reached. We hypothesize that PDOs remaining ‘viable’ at higher doses of RT might have become senescent, impeding further tumor growth. Further regrowth survival assays are necessary to investigate this hypothesis.

However, heterogeneity regarding response to irradiation between different PDOs remains present even with adequate time until readout and a sufficient irradiation dose. Pancreatic cancer is known for its heterogeneous biology, and it is likely that some patient derived PDOs respond better to treatment than others.[24] This emphasizes the importance to aim for patient-individualized therapies. To explain the diversity amongst pancreatic tumors, previous studies have examined their PDOs by whole exome sequencing, which revealed heterogeneous mutational patterns.[17], [18] Hu et al. investigated the resected specimens from six pancreatic cancer patients who underwent a resection followed by RT and compared the non-responders to the complete responders. Consequently, they identified SMAD3 and SMAD4 as potential biomarkers to predict the effectiveness of RT in pancreatic cancer.[25] These genetic variations and potential biomarkers should be the subject of further research, to gain a better understanding of inter- and intratumoral heterogeneity in pancreatic cancer.

Furthermore, PDOs could be useful to investigate combinatorial effects to improve treatment efficacy in individual patients. Nicosia et al. hypothesized that the application of RT with a magnetic field (MF) would enhance the efficacy of RT. They found that concurrent exposure to irradiation and MF reduced cell viability in all four PDOs tested. This effect was also verified on apoptotic cell death, which was increased in case of RT with MF in two out of three PDOs.[16] If a MF is indeed reinforcing the effect of radiation, this hypothetically could be an additional benefit of the application of MRgRT in clinical practice, aside from its increased precision for dose delivery. However, this theoretical benefit needs to be confirmed. Additionally, combined treatment mechanisms could enhance treatment effectiveness. Naumann et al. examined whether the combination of chemotherapy (gemcitabine or 5-fluorouracile (5FU)) and photon/proton RT increased pancreatic PDO response. Three PDOs were treated with 6 Gy of RT, 5FU, gemcitabine or a combination, and viability was assessed after 13 days. One of the PDOs showed a combined effect of 5FU and 6 Gy photon therapy, while another only showed a combined effect of 5FU or gemcitabine and 4 Gy proton therapy. The last PDO showed a combined effect of 5FU or gemcitabine and 6 Gy of proton therapy.[17] Although the observed results in the three examined PDOs were very heterogeneous, further investigation of combined effects between photon or photon therapy and different chemotherapy regimens could be promising. Lastly, the combination of immunotherapy and RT in pancreatic cancer could be beneficial, as RT can trigger the release of tumor-associated antigens and upregulate immune checkpoints, which are targeted by immunotherapy.[26], [27] However, to our knowledge, no studies on the combination of immunotherapy and RT in pancreatic cancer PDOs have been performed so far, and future studies on this topic should be conducted.

Although the experimental setup of this study was successful to evaluate the RT response in pancreatic cancer PDOs, this study also had shortcomings. First, only two PDOs were used, and a larger sample is desired to gain more certainty about the robustness of findings. Also, the viability percentage at certain radiation doses cannot be compared between different experiments because of the interexperimental variability in for example the concentration of organoids dissolved. Nevertheless, the goal of the experiments was to investigate the presence of a dose–response relation in the PDOs after radiotherapy and not necessarily to quantify the effect on survival, for which PDOs are suitable. In addition, drug testing in PDOs is frequently performed using viability assays, which captures ATP reduction due to reduced cell proliferation.[12] Although high-throughput radiation response assays closely mimic radiation response parameters of classic survival assays, additional alternative assessment such as the latter to assess regrowth of PDOs after RT should be evaluated in future studies.[12], [28], [29] In these experiments, organoids are treated with radiotherapy, after which they are washed and non-viable organoids are removed. Regrowth of the remaining organoids is longitudinally monitored to visualize progression over successive weeks. At the end of the experiment, a viability assay is executed to assess ATP levels, facilitating the quantitative evaluation of regrowth. Unfortunately, due to limited resources, this was not possible within the current study. Fourth, all experiments besides the final experiment were only performed once. However, the data points were acquired by performing viability assays in sextuple to avoid measurement errors. Next, the stroma of in vivo pancreatic cancer reaches up to 90 %.[30], [31] Some hypothesize that this protective desmoplastic stroma and immunosuppressive tumor microenvironment causes RT resistance in pancreatic cancer.[32], [33] Geyer et al. have established a pancreatic cancer model consisting of endothelial tube, pancreatic stellate cells (which when activated increase immune dysfunction and stimulate cancer cell invasion), and pancreatic cancer organoids. The investigators observed that stromal cells indeed create a physical barrier, preventing immune cells to migrate towards the pancreatic cancer cells, but also form a biochemical microenvironment that appears to attract and influence the distribution of immune cells.[34] Co-culture models of pancreatic cancer PDOs and cancer-associated fibroblasts have already been established, and future research should investigate the RT response in these pancreatic cancer PDO co-cultures, preferably with immune cells as well.[35], [36] Also, previous studies showed that pancreatic cancer PDOs of cachectic vs non-cachectic patients exhibit different patterns of cachexia-related factors.[37] Unfortunately, information on patients’ nutritional status was not available in the current study, but future experiments to evaluate the impact of cachexia on radiation response would be of interest. Lastly, it is not yet established whether the response of pancreatic cancer PDOs to radiation is a true reflection of the patient’s response. Shukla et al. compared the irradiation response of murine pancreatic cancer to their derived organoids and concluded that irradiation responses between both were comparable. Unirradiated murine pancreatic cancer and organoid size both increased in six-fold after 10 days, showing similar proliferation. However, dose levels of 4 and 6 Gy did impede growth, and at a dose level of 8 Gy growth was fully stagnated.[38] Despite the fact that irradiation responses of murine pancreatic cancer might differ from human pancreatic cancer, this study suggests that PDOs are a representative model for the in vivo tumor. Nevertheless, future research should correlate *in vitro* organoid responses to corresponding patient outcomes before organoid based personalized medicine can be implemented in clinical settings.

In conclusion, this study confirms that PDOs can be used to model radiation response in pancreatic cancer. Pancreatic cancer PDOs are sensitive to radiation, which has been contradicted in previous literature, although sufficient time to readout is crucial for proper evaluation of results.

## Informed consent

Informed consent was obtained from all subjects involved in the study.

## Data availability

All data created was made available in this manuscript.

## Funding

None.

## Declaration of competing interest

The authors declare that they have no known competing financial interests or personal relationships that could have appeared to influence the work reported in this paper.
