# Supplementary material for: Radiation response assessment of organoids derived from patients with pancreatic cancer
Source: Clin Transl Radiat Oncol. 2024 Jul 27;48:100829. doi: 10.1016/j.ctro.2024.100829 (PMC11347840; doi:10.1016/j.ctro.2024.100829)
Supplement: Supplementary Data 1 [file mmc1.docx]

**Appendices**

| **Supplementary table 1a. Genetic alterations in HUB-08-B2-022A^$^** | |
| --- | --- |
| **Gene** | **Effect** |
| KRAS | Missense |
| TP53 | Missense, loss of heterozygosity |
| CDKN2A | Deep deletion |
| SMAD4 | Deep deletion |
| ARID1A | Missense |
| KDM6A | Deep deletion |
| * Assuming UPE Noggin is used. If recombinant Noggin is used, supplement with an additional 38 mL of Ad-DF+++.  ^$^ Results obtained by Driehuis et al.^18^ | |

| **Supplementary table 1b. Genetic alterations in HUB-08-B2-026B^$^** | |
| --- | --- |
| **Gene** | **Effect** |
| KRAS | Missense |
| TP53 | Missense, loss of heterozygosity |
| CDKN2A | Deep deletion |
| EEF2 | Missense |
| SMAD4 | Deep deletion |
| GNAS | Amplification |
| ^$^ Results obtained by Driehuis et al.^18^ | |

| **Supplementary table 2a. Pancreatic base medium (PBM)^$^** | | |
| --- | --- | --- |
| **Supplement** | **Company** | **Concentration** |
| Ad-DF+++ | Not applicable | 920* |
| B27 (50x) | 2x | 40 |
| *UPE Noggin (100%) or recombinant Noggin (100 µg/mL) | 4% UPE Noggin or 200 ng/mL recombinant Noggin | 40 or 2, respectively |
| N-Acetyl Cysteine | 2.5 mM | 0.41 gram dissolved in 5 mL H_2_O |
| Nicotinamide | 20 mM | 2.44 gram dissolved in 5 mL H_2_O |
| Gastrin (100 µM) | 20 nM | 0.2 |
| **Total** |  | **1,000** |
| * Assuming UPE Noggin is used. If recombinant Noggin is used, supplement with an additional 38 mL of Ad-DF+++.  ^$^ As described by Driehuis et al.^18^ | | |

| **Supplementary table 2b. Pancreatic tumor medium 1 (TM1)^$^** | | |
| --- | --- | --- |
| **Supplement** | **Company** | **Concentration** |
| Pancreatic base medium | 1 | 50 mL |
| *Wnt CM or Wnt surrogate | 0.5x or 0.5 nM* | 50 mL or X µL* |
| Recombinant R-spondin 3 (conc. varies depending on the stock) | 250 ng/mL | Varies depending on the stock |
| Primocin (50 mg/mL) | 50 µg/mL | 100 µL |
| FGF10 (100 µg/mL) | 100 ng/mL | 100 µL |
| A83-01 (5 mM) | 500 nM | 10 µL |
| **Total** |  | **100 mL** |
| * Wnt surrogate has varying concentrations depending on the batch. Therefore, the final volume that is required to be added differs between different batches used. If Wnt surrogate is used as a Wnt source instead of Wnt CM, supplement with an additional 50 mL of Ad-DF+++ to reach the total volume of 100 mL pancreatic tumor medium 1.  ^$^ As described by Driehuis et al.^18^ | | |

| **Supplementary table 2c. Pancreatic tumor medium 2 (TM2)^$^** | | |
| --- | --- | --- |
| **Supplement** | **Company** | **Concentration** |
| Pancreatic base medium | 1 | 50 mL |
| Ad-DF+++ | Not applicable | 50 mL |
| Recombinant R-spondin 3 (conc. varies depending on the stock) | 250 ng/mL | Varies depending on the stock |
| Primocin (50 mg/mL) | 50 µg/mL | 100 µL |
| EGF (50 µg/mL) | 50 ng/mL | 100 µL |
| FGF10 (100 µg/mL) | 100 ng/mL | 100 µL |
| **Total** |  | **100 mL** |
| ^$^ As described by Driehuis et al.^18^ | | |

| a HUB-08-B2-022A  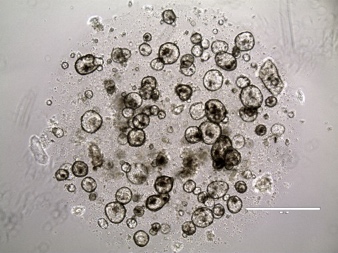  4x | b HUB-08-B2-026B  *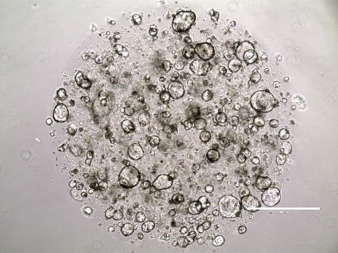*  4x |
| --- | --- |
| 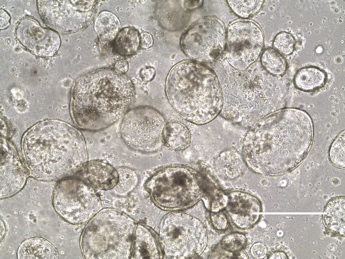  10x | *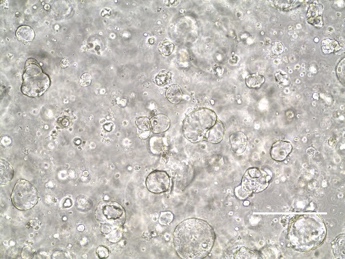*  10x |
| 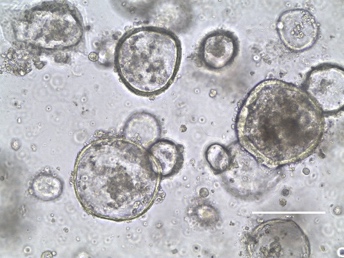  20x | *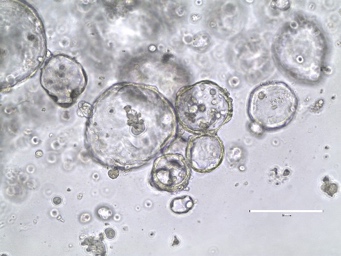*  20x |
| 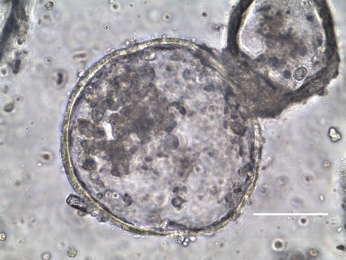  40x | *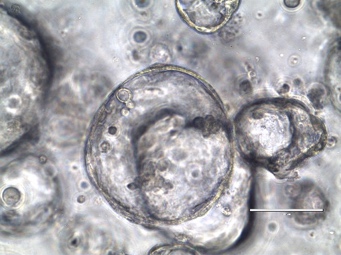*  40x |
| *Supplemental figure 1: Organoids in culture.*  *a: HUB-08-B2-22A in culture shown in four magnifications, b: HUB-08-B2-26B in culture shown in four magnifications.* | |
